# Validation of the Finnish Diabetes Risk Score and development of a country-specific diabetes prediction model for Turkey

**DOI:** 10.1017/S1463423625000180

**Published:** 2025-02-26

**Authors:** Neslisah Ture, Ahmet Naci Emecen, Belgin Unal

**Affiliations:** 1 Ayvacik District of Health Directorate, Canakkale, Turkey; 2 Faculty of Medicine, Department of Public Health, Epidemiology Subsection, Dokuz Eylul University, Izmir, Turkey

**Keywords:** diabetes mellitus, primary prevention, risk assessment

## Abstract

**Aims:**

Diabetes is a global health concern, and early identification of high-risk individuals is crucial for preventive interventions. Finnish Diabetes Risk Score (FINDRISC) is a widely accepted non-invasive tool that estimates the 10-year diabetes risk. This study aims to validate the FINDRISC in the Turkish population and develop a specific model using data from a nationwide cohort.

**Method:**

The study used data of 12249 participants from the Türkiye Chronic Diseases and Risk Factors Survey. Data included sociodemographic variables, lifestyle factors, and anthropometric measurements. Multivariable logistic regression was employed using FINDRISC variables to predict incident type 2 diabetes mellitus (T2DM). Two country-specific models, one incorporating the waist-to-hip ratio (WHR model) and the other waist circumference (WC model), were developed. The least absolute shrinkage and selection operator (LASSO) algorithm was used for variable selection in the final models, and model discrimination indexes were compared.

**Results:**

The optimal FINDRISC cut-off was 8.5, with an area under the curve (AUC) of 0.76, demonstrating good predictive performance in identifying T2DM cases in the Turkish population. Both WHR and WC models showed similar predictive accuracy (AUC: 0.77). Marital status and education were associated with increased diabetes risk in both country-specific models.

**Conclusion:**

The study found that the FINDRISC tool is effective in predicting the risk of type 2 diabetes in the Turkish population. Models using WHR and WC showed similar predictive performance to FINDRISC. Sociodemographic factors may play a role in diabetes risk. These findings highlight the need to consider population-specific characteristics when evaluating diabetes risk.

## Introduction

Diabetes is one of the significant causes of mortality and morbidity all around the world. The increasing prevalence of type 2 diabetes mellitus (T2DM) is accelerating the burden on healthcare systems (Safiri *et al.*, 2022; GBD 2021 Diabetes Collaborators, 2023). Numerous studies have shown that identifying individuals at high risk for diabetes and implementing lifestyle interventions can significantly reduce the risk of developing diabetes. In Finland, the Finnish Diabetes Risk Score (FINDRISC) has been used in primary healthcare, pharmacies, and nationwide advertising campaigns to identify high-risk individuals. Targeted interventions for these individuals led to a 58% reduction in diabetes incidence within 3 years (Saaristo *et al.*, 2010; Rintamäki *et al.*, 2021).

In the context of primary prevention, various strategies have been employed to screen for diabetes risk factors and identify individuals at high risk within the general population. While the American Diabetes Association recommends fasting blood glucose (FBG) and oral glucose tolerance tests (OGTTs) to detect high-risk individuals and those with asymptomatic diabetes (American Diabetes Association Professional Practice Committee, 2021), screening the entire population using these methods is challenging, time-consuming, and not cost-effective. As a result, there has been a shift towards more cost-effective and non-invasive risk assessment tools that can be easily applied in primary care settings (Stern *et al*., 2002; Fagg and Valabhji, 2019). Hence, various predictive risk scores have been developed to assess an individual’s risk of diabetes.

FINDRISC is one of the widely recommended and internationally accepted non-invasive risk assessment tools. It estimates an individual’s likelihood of developing diabetes over a 10-year period by considering eight diabetes-associated predictors. The primary objective of this score is to identify individuals at high risk of diabetes and provide them lifestyle counselling to prevent the early onset of diabetes and reduce the likelihood of associated complications (Lindström and Tuomilehto, 2003). Since countries differ in the distribution of risk factors, lifestyle, and genetic composition, it is necessary to assess the applicability of these risk scores within specific populations (Glümer *et al*., 2005). FINDRISC score has been validated in many European countries, including Sweden (Hellgren *et al.*, 2012), Greece (Makrilakis *et al.*, 2011), Spain (Soriguer *et al.*, 2012), Italy (Franciosi *et al.*, 2005), Bulgaria (Tankova *et al.*, 2011), and Slovenia (Štiglic *et al.*, 2016).

In this study, we aimed to validate the FINDRISC questionnaire in Turkish population and to investigate the development of country-specific model for Turkish people.

## Method

### Study design and data source

This retrospective cohort study utilized data from the Türkiye Chronic Diseases and Risk Factors Survey (TurCDRFs) (Ünal *et al.*, 2013) to evaluate the applicability of the FINDRISC in the Turkish population. The TurCDRF survey was a nationwide effort conducted by the Turkish Ministry of Health in 2011 to determine the prevalence of chronic diseases and associated risk factors. Data were collected by family physicians (FPs) from their registered populations, aged 15 years and older. Participants were invited to Family Health Centers (FHCs) for face-to-face interviews, physical examinations, and laboratory tests. The data were then entered into an electronic system (Ünal *et al.*, 2013).

The participants of the 2011 survey were electronically tracked through national databases until 2017 to monitor the development of chronic diseases, hospitalizations, medical prescriptions, and survival outcomes (Kara *et al.*, 2021). The national data sources linked to baseline 2011 database included the Social Security Institution (SSI) and the National Death Notification System (NDNS). The SSI covers health expenses in Türkiye, including treatments, medicines, laboratory, and imaging tests. The NDNS records causes of death using ICD-10 codes, as determined by physicians.

### Study population and diabetes diagnosis

The initial dataset consisted of 18477 participants aged 15 years and older in 2011. After excluding 4997 individuals with a prior history of T2DM and 1231 with missing data on FINDRISC variables, a total of 12249 participants were included in the study. Participants who were initially healthy in 2011 and were diagnosed with T2DM based on ICD-10 codes (E11–E14) at least twice during the 6-year follow-up period were classified as incident T2DM cases. Single entries of ICD-10 codes (E11–E14) were not considered sufficient for T2DM classification unless the individual had been hospitalized, received treatment, or had diabetes listed as a cause of death. Individuals with diabetes listed as the cause of death were also classified as incident cases.

### Data collection and preparation for the analysis

FINDRISC was developed to identify high-risk individuals for diabetes and predict their 10-year diabetes risk using a questionnaire with eight measures: age (categorized, years), BMI (categorized, kg/m^2^), waist circumference (WC) (categorized, cm), physical activity (>30 minutes/day), daily intake of fruits/vegetables/berries, history of medication for hypertension, self-reported history of high blood glucose, and family history of diabetes. Scores on the FINDRISC questionnaire range from 0 to 26 points, with risk categories typically classified as low (0–6 points), slightly elevated (7–11 points), moderate (12–14 points), high (15–20 points), or very high (21–26 points). Higher scores indicate a greater risk of developing diabetes, making FINDRISC a valuable tool for individualized preventive interventions (Lindström and Tuomilehto, 2003; Saaristo *et al.*, 2005).

In the baseline TurCDRF survey, sociodemographic characteristics, lifestyle factors, and both family and personal medical histories were collected by FPs. Additionally, anthropometric measurements, including weight, height, and waist and hip circumference, were taken. Sociodemographic characteristics included age, gender, educational level, place of residence, and marital status. Age was categorized into four groups (<45, 45–54, 55–64, and ≥ 65 years) in accordance with the FINDRISC questionnaire. Educational level was classified into two categories: less than high school and high school or university. Place of residence was categorized as urban for populations exceeding 20000 and rural for populations of 20000 or less.

Lifestyle factors included smoking status, alcohol use, physical activity, type of bread consumed, type of oil/butter consumed, and additional salt intake. Smoking status was categorized as current smoking (regular or occasional) or not (non-smoker or ex-smoker). In the original TurCDRF dataset, physical activity was assessed in terms of intensity, frequency, and duration. Participants were categorized into three groups: adequate physical activity (engaging in moderate or vigorous activity at least five times a week for 30 minutes), moderate physical activity (1–4 times a week for 30 minutes), and low physical activity (less than once a week for 30 minutes or none). In alignment with the FINDRISC questionnaire, ‘Yes’ to engaging in 30 minutes of daily exercise was considered ‘adequate’, while ‘No’ was classified as ‘moderate to low’ physical activity. Daily consumption of vegetables and fruits was recorded as either ‘0 portions’ (no daily consumption) or ‘one or more portions’ (daily consumption). Participants reported their bread preferences, including white, whole-wheat, rye, and oat bread. For the type of oil/butter consumed, options included butter, margarine, olive oil, and other types such as sunflower, corn, soy, or hazelnut. Salt intake habits were assessed based on whether participants added salt to their meals at the dining table without tasting first. Participants’ weight, height, and WC were also measured by FPs during data collection in 2011.

Data on personal and family medical histories were based on self-report. Hypertension was assessed with the questions, ‘Have you ever been told by a physician that you have high blood pressure?’ and ‘Do you use any drugs regularly for this condition?’ (yes/no). Some variables in our database were reconstructed to align with the FINDRISC scoring system. For example, the FINDRISC questionnaire assesses a history of high blood glucose with the question, ‘Have you ever been found to have high blood glucose?’ (e.g., in a health examination, during an illness, or during pregnancy). In our study, this question was not self-reported; instead, individuals with impaired FBG levels detected through blood measurements taken in 2011 were considered to have answered ‘yes’. Additionally, while the FINDRISC questionnaire assigns different points for first-degree and second-degree relatives in the family history of diabetes, our study considered only first-degree relatives.

### Statistical analysis

Categorical data were presented as number and percentages (n, %). In the comparison of categorical variables between incident T2DM group and the people who did not develop T2DM, Pearson’s chi-square test with Yates’ continuity correction was used. Receiver operating characteristics (ROC) curve analysis was used to find the optimal cut-off (Method: Youden index) of continuous FINDRISC score in the prediction of T2DM. For the external validation of FINDRISC in Turkish population, a multivariable logistic regression model with FINDRISC variables was built. To develop a country-specific model, we generated two separate models due to highly correlation between waist-to-hip ratio (WHR) and WC: WHR model and WC model. The least absolute shrinkage and selection operator (LASSO) algorithm was utilized to select potential variables that might impact the diagnosis of T2DM. In addition to FINDRISC variables, candidate variables for WHR model were gender, place of residence, education, marital status, current smoking, alcohol use, type of bread consumed, type of oil/butter consumed, additional salt intake, and WHR. For WC model, WHR was replaced by WC. The optimal regularization parameter (λ) was estimated by 10-fold cross-validation. Prior to selection procedure, missing data on place of residence, education, marital status, current smoking, alcohol use, type of bread consumed, type of oil/butter consumed, additional salt intake, and WHR were imputed using logistic regression or polytomous logistic regression. Discrimination indexes of three models (area under the curve [AUC] and Brier index) were compared. Risk measure was odds ratio (OR) with its 95% confidence interval (95% CI). Statistical analysis and visualizations were performed with R version 4.3.1 (A language and environment for statistical computing. R Foundation for Statistical Computing, Vienna, Austria. https://www.R-project.org/).

### Ethical considerations

An official permission to use anonymized data was obtained from the Ministry of Health of Turkiye (Date: 28.08.2022 No: E-96867468-151.01). The study was approved with decision number 2022/30-15 dated September 21, 2022, by Non-Interventional Research Ethics Committee of Dokuz Eylul University.

## Results

The study included a total of 12249 participants, of whom 505 developed incident T2DM during the study period. The 6-year cumulative incidence was 4122.8 per 100000 people aged ≥15 years. Among individuals in the high and very high-risk groups, the proportion developing T2DM was 10.9% and 17.8%, respectively. The optimal FINDRISC cut-off score for predicting T2DM was found to be 8.5 (AUC, 95% CI: 0.75, 0.73–0.77; sensitivity: 78.4%, specificity: 59.0%, positive predictive value (PPV): 7.6%, negative predictive value (NPV): 98.5%).

**Figure 1.**
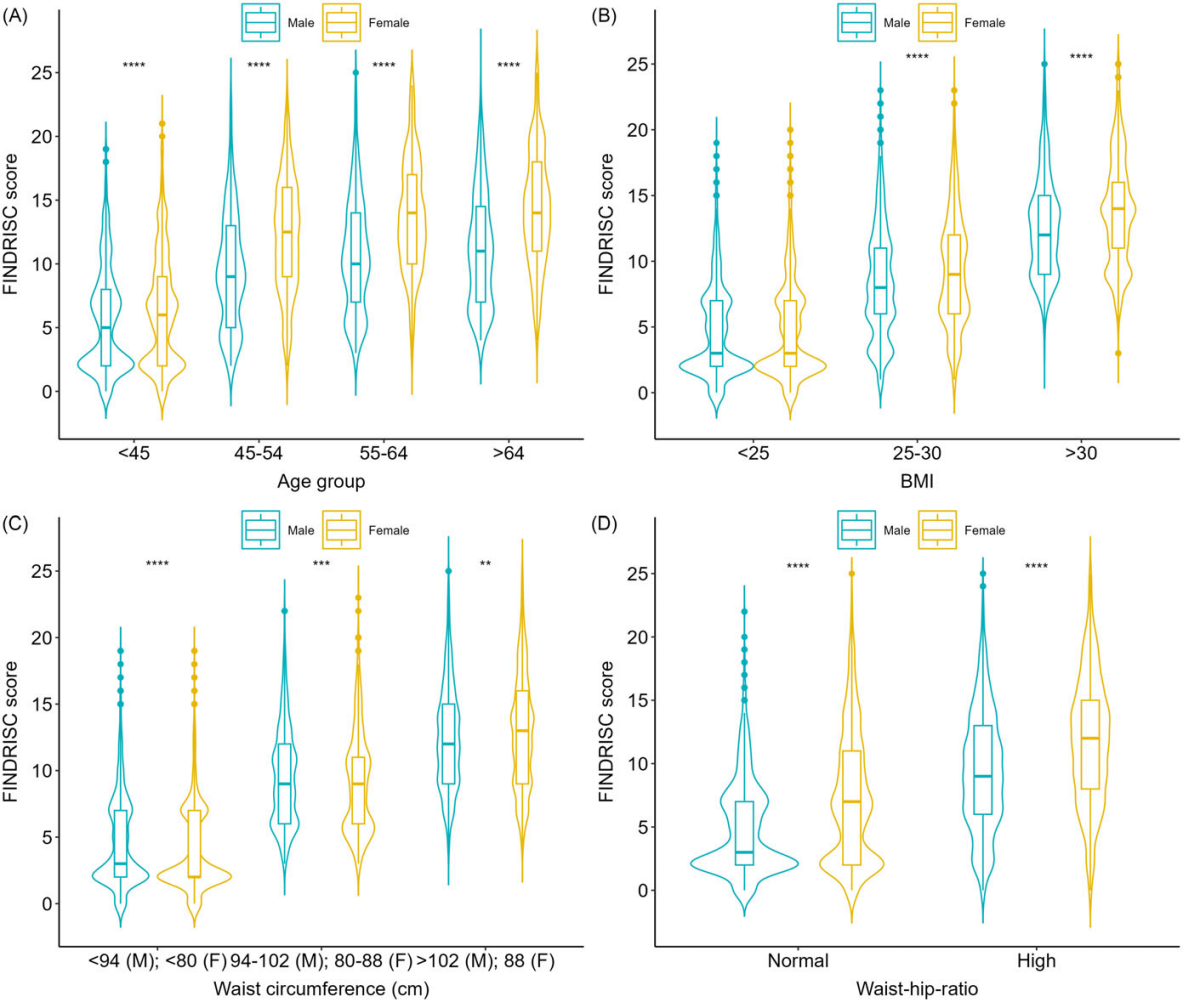
Violin plots of FINDRISC scores for age group (A), BMI (B), waist circumference (C), and waist-to-hip ratio (D), stratified by gender. Comparisons by gender are labelled as follows: **p ≤ 0.01, ***p ≤ 0.001, ****p ≤ 0.0001.

Figure 1 presents violin plots illustrating the distribution of FINDRISC scores for age group, BMI, WC, and WHR, further stratified by gender. The plots reveal that FINDRISC scores were generally lower in younger age groups, with the distribution widening as age increased, reflecting greater variability in diabetes risk among older individuals. In the older age categories, females showed higher risk scores. Additionally, median FINDRISC scores increased with higher BMI categories. Scores were also higher in categories with larger WC and elevated WHR, with females exhibiting higher scores in these categories.

Table 1 presents descriptive characteristics of the study population and incident T2DM cases by groups. A higher proportion of females (4.7%) developed T2DM compared to males (3.4%). The incidence of T2DM also increased with age, with the highest rates observed in individuals aged 55–64 years (8.1%) and those over 64 years (7.2%). Additionally, participants with a family history of diabetes, elevated blood glucose levels, higher WC, high WHR, and elevated BMI were more likely to develop T2DM.

**Table 1.** General characteristics of the population included in the study and incident type 2 diabetes mellitus cases by groups

	N	All, n (%*)	Incident DM, n = 505, (%**)	p
**Gender**	12249			<0.001
Male		5796 (47.3)	199 (3.4)	
Female		6453 (52.7)	306 (4.7)	
**Age category (years)**	12249			<0.001
<45		7981 (65.2)	184 (2.3)	
45–54		1837 (15.0)	134 (7.3)	
55–64		1282 (10.5)	104 (8.1)	
>64		1149 (9.3)	83 (7.2)	
**Place of residence**	12070			0.204
Rural		3830 (31.7)	144 (3.8)	
Urban		8240 (68.3)	352 (4.3)	
**Education**	12244			0.133
Less than high school		8767 (71.6)	377 (4.3)	
High school–university		3477 (28.4)	128 (3.7)	
**Marital status**	12242			<0.001
Single		3734 (30.5)	86 (2.3)	
Married		8508 (69.5)	418 (4.9)	
**Family history of DM**	12249			<0.001
No		9000 (73.5)	320 (3.6)	
Yes		3249 (26.5)	185 (5.7)	
**Current smoking**	12217			0.02
Yes		3651 (29.9)	127 (3.5)	
No		8566 (70.1)	378 (4.4)	
**Alcohol use**	12178			0.187
No		10501 (86.2)	447 (4.3)	
<5 per month		1398 (11.5)	50 (3.6)	
>1 per week		279 (2.3)	7 (2.5)	
**Antihypertensive medication use**	12249			<0.001
No		10655 (87.0)	327 (3.1)	
Yes		1594 (13.0)	178 (11.2)	
**Type of bread consumed**	12143			<0.001
White		10689 (88.0)	411 (3.9)	
Other		1454 (12.0)	85 (5.9)	
**Type of oil/butter consumed**	12200			0.003
Butter		863 (7.1)	27 (3.1)	
Margarine		356 (2.9)	9 (2.5)	
Olive oil		3506 (28.7)	178 (5.1)	
Other		7475 (61.3)	290 (3.9)	
**Additional salt intake**	12112			0.230
Yes		2246 (18.5)	82 (3.7)	
No		9866 (81.5)	418 (4.2)	
**Intake of vegetables/fruits/berries**	12249			0.283
Everyday		11761 (96.0)	490 (4.2)	
Not everyday		488 (4.0)	15 (3.1)	
**BMI category**	12249			<0.001
<25		5435 (44.4)	91 (1.7)	
25–30		4076 (33.3)	163 (4.0)	
>30		2738 (22.3)	251 (9.2)	
**Waist circumference (cm)**	12249			<0.001
<94 (M); <80 (F)		5807 (47.4)	89 (1.5)	
94–102 (M); 80–88 (F)		2727 (22.3)	108 (4.0)	
>102 (M); 88 (F)		3715 (30.3)	308 (8.3)	
**Waist-to-hip ratio**	12203			<0.001
Normal		7169 (58.7)	192 (2.7)	
High		5034 (41.3)	312 (6.2)	
**Physical activity**	12249			0.561
Yes		2221 (18.1)	97 (4.4)	
No		10028 (81.9)	408 (4.1)	
**High blood glucose**	12249			<0.001
No		10223 (83.5)	340 (3.3)	
Yes		2026 (16.5)	165 (8.1)	
**FINDRISC risk category**	12249			<0.001
Low		5159 (42.1)	59 (1.1)	
Slightly elevated		3985 (32.5)	152 (3.8)	
Moderate		1641 (13.4)	123 (7.5)	
High		1301 (10.6)	142 (10.9)	
Very high		163 (1.4)	29 (17.8)	

*Over total n, **row percentage.

Results from the multivariable logistic regression model, which included only FINDRISC variables, are summarized in Table 2. Age was a significant predictor across all age subgroups. Participants with a BMI over 30 kg/m^2^ had significantly higher odds of developing T2DM (OR: 1.94, 95% CI: 1.36–2.77). A WC exceeding 102 cm in males or 88 cm in females was also strongly associated with an increased risk of T2DM (OR: 2.22, 95% CI: 1.57–3.15). Additionally, the use of antihypertensive medication (OR: 1.91, 95% CI: 1.52–2.38), high blood glucose levels (OR: 1.80, 95% CI: 1.47–2.19), and a family history of diabetes (OR: 1.43, 95% CI: 1.18–1.73) were significantly associated with a higher likelihood of developing T2DM (Table 2).

**Table 2.** Univariate and multivariate logistic regression models for type 2 diabetes mellitus with FINDRISC variables

	Univariate (OR, 95% CI)	Multivariate (aOR, 95% CI)
**Age category (years)**		
<45	Ref.	Ref.
45–54	3.33 (2.65–4.19)	1.86 (1.46–2.38)
55–64	3.74 (2.91–4.78)	1.82 (1.38–2.40)
>64	3.30 (2.52–4.29)	1.64 (1.21–2.22)
**BMI category**		
<25 kg/m^2^	Ref.	Ref.
25–30 kg/m^2^	2.45 (1.89–3.18)	1.29 (0.95–1.77)
>30 kg/m^2^	5.93 (4.66–7.60)	1.94 (1.36–2.77)
**Waist circumference (cm)**		
<94 (M); <80 (F)	Ref.	Ref.
94–102 (M); 80–88 (F)	2.65 (2.00–3.53)	1.73 (1.25–2.40)
>102 (M); 88 (F)	5.81 (4.59–7.42)	2.22 (1.57–3.15)
**Physical activity**		
Yes	Ref.	Ref.
No	0.93 (0.74–1.17)	0.96 (0.77–1.22)
**Intake of vegetables/fruits/berries**		
Everyday	Ref.	Ref.
Not everyday	0.73 (0.41–1.19)	0.87 (0.49–1.43)
**Antihypertensive medication use**		
No	Ref.	Ref.
Yes	3.97 (3.28–4.80)	1.91 (1.52–2.38)
**High blood glucose**		
No	Ref.	Ref.
Yes	2.58 (2.12–3.12)	1.80 (1.47–2.19)
**Family history of DM**		
No	Ref.	Ref.
Yes	1.64 (1.36–1.97)	1.43 (1.18–1.73)

BMI: body mass index, DM: diabetes mellitus, OR: odds ratio, aOR: adjusted odds ratio.

LASSO regression was used to identify potential predictors of diabetes in the Turkish population, incorporating models separately using WHR and WC, along with other study variables. In both the WHR and WC models, significant predictors included age category, education, marital status, family history of diabetes, antihypertensive medication use, BMI >30 kg/m^2^ category, and high blood glucose levels. The ORs (95% CI) for each variable are presented in Table 3. High WHR was significantly associated with an increased risk of T2DM (OR: 1.40, 95% CI: 1.14–1.73), while being in a high WC category (>102 cm for males, >88 cm for females) was also a strong predictor of T2DM (OR: 2.21, 95% CI: 1.57–3.13) in the WC model.

**Table 3.** Prediction models with selected variables for identifying type 2 diabetes mellitus in Turkish population

	Univariate OR, 95% CI	WHR model aOR, 95% CI	WC model aOR, 95% CI
**Gender**			Not selected
Male	Ref.	Ref.	
Female	1.40 (1.17–1.68)	1.27 (1.02–1.58)	
**Age category (years)**			
<45	Ref.	Ref.	Ref.
45–54	3.33 (2.65–4.19)	1.93 (1.50–2.47)	1.89 (1.47–2.42)
55–64	3.74 (2.91–4.78)	2.00 (1.50–2.64)	1.94 (1.46–2.56)
>64	3.30 (2.52–4.29)	1.97 (1.43–2.70)	1.94 (1.41–2.65)
**Place of residence**			
Rural	Ref.	Ref.	Ref.
Urban	1.15 (0.95–1.40)	1.15 (0.94–1.42)	1.15 (0.93–1.42)
**Education**			
Less than high school	Ref.	Ref.	Ref.
High school–university	0.85 (0.69–1.04)	1.51 (1.19–1.90)	1.50 (1.19–1.88)
**Marital status**			
Single	Ref.	Ref.	Ref.
Married	2.20 (1.75–2.80)	1.52 (1.19–1.97)	1.48 (1.16–1.92)
**Family history of DM**			
No	Ref.	Ref.	Ref.
Yes	1.64 (1.36–1.97)	1.41 (1.16–1.71)	1.40 (1.16–1.70)
**Alcohol use**			
No	Ref.	Ref.	Ref.
<5 per month	0.83 (0.61–1.11)	1.09 (0.78–1.49)	1.07 (0.78–1.46)
>1 per week	0.58 (0.25–1.14)	0.57 (0.24–1.16)	0.56 (0.23–1.12)
**Antihypertensive medication use**			
No	Ref.	Ref.	Ref.
Yes	3.97 (3.28–4.80)	1.93 (1.54–2.42)	1.93 (1.54–2.41)
**Type of oil/butter consumed**			
Butter	Ref.	Ref.	Ref.
Margarine	0.81 (0.35–1.67)	0.91 (0.39–1.90)	0.91 (0.40–1.91)
Olive oil	1.66 (1.12–2.55)	1.43 (0.95–2.23)	1.43 (0.95–2.22)
Other	1.26 (0.86–1.92)	1.23 (0.83–1.90)	1.24 (0.84–1.91)
**BMI category**			
<25 kg/m^2^	Ref.	Ref.	Ref.
25–30 kg/m^2^	2.45 (1.89–3.18)	1.59 (1.21–2.09)	1.23 (0.90–1.68)
>30 kg/m^2^	5.93 (4.66–7.60)	2.90 (2.20–3.84)	1.90 (1.34–2.72)
**Waist-to-hip ratio (WHR)**			Not included
Normal	Ref.	Ref.	–
High	2.41 (2.01–2.89)	1.40 (1.14–1.73)	–
**Waist circumference (cm)**		Not included	
<94 (M); <80 (F)	Ref.	–	Ref.
94–102 (M); 80–88 (F)	2.65 (2.00–3.53)	–	1.66 (1.20–2.30)
>102 (M); 88 (F)	5.81 (4.59–7.42)	–	2.21 (1.57–3.13)
**High blood glucose**			
No	Ref.	Ref.	Ref.
Yes	2.58 (2.12–3.12)	1.83 (1.49–2.24)	1.82 (1.49–2.23)

Variable selection process was based on the least absolute shrinkage and selection operator (LASSO) algorithm.

The potential variables for the WHR model included gender, age category, place of residence, education, marital status, family history of diabetes, current smoking, alcohol use, antihypertensive medication use, type of bread consumed, type of oil/butter consumed, additional salt intake, intake of vegetables/fruits/berries, BMI category, WHR, and high blood glucose.

The potential variables for the WC model were the same as those in the WHR model, with the substitution of waist circumference for the WHR.

**Figure 2.**
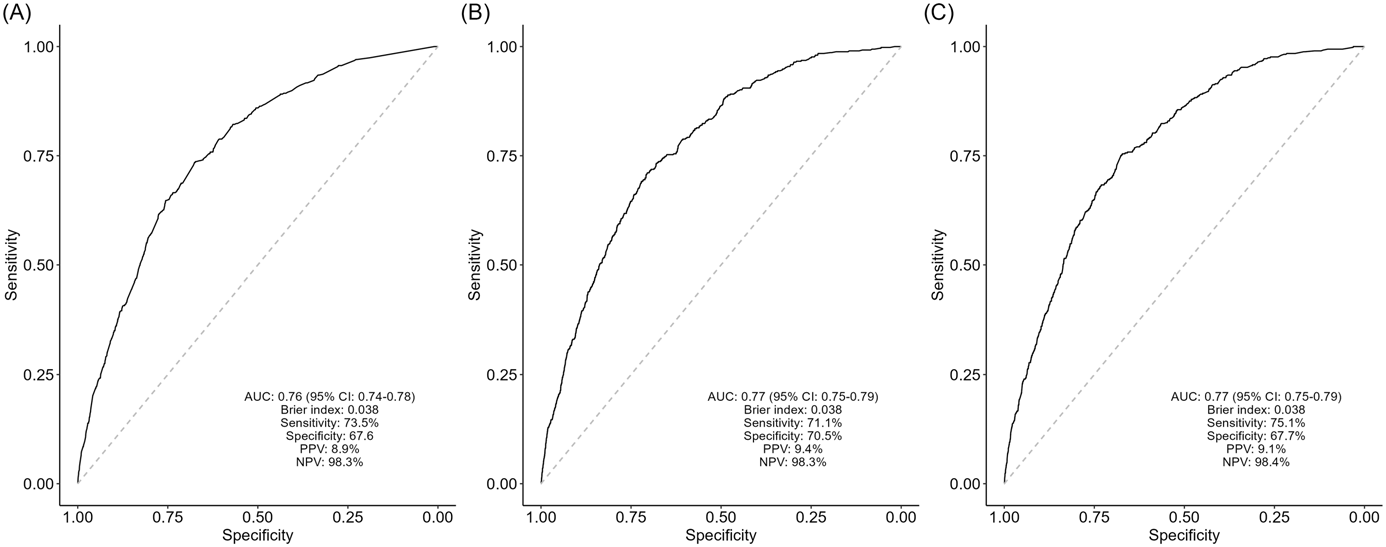
Receiver operating characteristic (ROC) curve analysis of predicted probabilities for type 2 diabetes mellitus, with model discrimination indexes. Multivariable model with only FINDRISC variables (A), waist-to-hip ratio (WHR) model with LASSO-selected variables (B), and waist circumference (WC) model with LASSO-selected variables (C).

The FINDRISC score demonstrated good performance in predicting T2DM within a 6-year period, with an AUC of 0.76 (95% CI: 0.74–0.78). Both the WHR and WC models showed similar predictive performance, with an AUC of 0.77 (95% CI: 0.74–0.79) and a Brier score of 0.038 for both models (Figure 2).

## Discussion

In this study, the performance of FINDRISC score was demonstrated well in Turkish population, showing an AUC of 0.76 (sensitivity: 73.5%, specificity: 67.6%). When comparing the performance of the FINDRISC score across various populations, previous studies have reported AUC values ranging from 0.63 to 0.87 (Lindström and Tuomilehto, 2003; Omech *et al.*, 2016; Lim *et al*., 2020). These values generally fell below the performance observed in the Finnish population (AUC: 0.85). However, FINDRISC demonstrated similar performance in the majority of studies, including Malaysia (AUC: 0.76) (Lim *et al*., 2020), Spain (AUC: 0.75) (Salinero-Fort *et al.*, 2016), Norway (AUC: 0.77) (Jølle *et al.*, 2019), and a European cohort (AUC: 0.74) (Gabriel *et al.*, 2021).

Two separate county-specific models, one incorporating WHR and the other incorporating WC, also demonstrated good performance in predicting 6-year T2DM, each with an AUC of 0.77. The WHR model had a sensitivity of 71.1% and specificity of 70.5%, while the WC model had a sensitivity of 75.1% and specificity of 67.7%. A study from China developed a simplified prediction model for Chinese population and found an AUC of 0.67 (sensitivity: 84.2%, specificity: 39.8%), while FINDRISC score showed an AUC of 0.66. In contrast to our findings, both the Chinese simplified diabetes risk score and the FINDRISC score demonstrated relatively lower performance (Gao *et al.*, 2010). This difference may be attributed to variations in population characteristics, study designs – often involving cross-sectional studies – and sample sizes.

In this study, we found similar performance between the FINDRISC and country-specific models in predicting T2DM within the Turkish population. Therefore, both the FINDRISC score and our models appear suitable for determining diabetes risk in the Turkish population. When estimating diabetes risk, commonly considered predictors include age, gender, BMI, WC, physical activity, dietary habits, hypertension, and a family history of diabetes (Lindström and Tuomilehto, 2003). However, we expanded on this by incorporating sociodemographic factors such as marital status, educational level, and place of residence, along with additional lifestyle-related variables like smoking, alcohol consumption, type of bread consumed, type of oil/butter consumed, and additional salt intake.

In a meta-analysis comparing the predictive abilities of BMI, WC, and WHR for diabetes incidence, it was found that despite the high correlation between BMI and WC, and the relatively low correlation of WHR with these measures, all three variables showed similar predictive performance in estimating diabetes incidence (Vazquez *et al.*, 2007). In our study, we observed that WC (94–102 (M); 80–88 (F), OR: 1.66, 95% Cl: 1.20–2.30; >102 (M); 88 (F) OR: 2.21, 95% Cl: 1.57–3.13) contributed to a higher increase in diabetes risk compared to WHR (OR: 1.40, 95% Cl: 1.14–1.73). However, when we assessed the overall performance of the models, both WC and WHR models demonstrated equivalent predictive power.

Our findings indicated that being married and having a higher level of education were associated with an increased risk of diabetes. The relationship between marriage and diabetes is complex. Previous studies have shown that the risk of diabetes was higher in single, divorced men, and widowed women (Kposowa *et al*., 2021). However, in our study, the increased risk among married individuals may be explained by the possibility that spouses provide support, leading to better healthcare access and higher diagnosis rates among married individuals.

In the literature, lower educational levels are often associated with an increased risk of diabetes (Sezer *et al.*, 2021). However, our study found that individuals with higher educational background had a higher risk of developing diabetes. A study conducted in the USA also found that higher educational levels were associated with an increased the risk of T2DM (Zhang *et al.*, 2014). This paradoxical finding may be attributed to the fact that individuals with higher education are more aware of health issues and may seek medical care more frequently, leading to higher diagnosis rates. Therefore, targeted interventions may be needed for disadvantaged groups, such as those with lower educational attainment and participants with low social support.

In this study, multivariable logistic regression analysis was conducted in externally validate FINDRISC variables. Both physical activity (OR: 0.96, 95% Cl: 0.77–1.22) and daily intake of fruit and vegetables (OR: 0.87, 95% Cl: 0.49–1.43) were not statistically significant predictors of T2DM. Simplified versions of the FINDRISC model have been evaluated and found to perform similarly or better in different populations. For instance, simplified versions of FINDRISC were successfully implemented in Spain (Salinero-Fort *et al.*, 2016) (BMI, history of high blood glucose, and antihypertensive medication use), an European cohort (Mavrogianni *et al.*, 2019) (age, BMI, antihypertensive medication use, and history of high blood glucose), and Burkina Faso (Traoré *et al.*, 2021) (age, daily physical activity, antihypertensive medication use, WC, and BMI). These results indicate that there may be additional factors or modifications specific to our population that should be integrated into the risk assessment model for more accurate predictions.

This is the first study to evaluate FINDRISC performance in Turkish population using a large cohort. Additionally, it contributes to the development of a diabetes risk score specifically modelled for the Turkish population. However, there are several limitations to consider. First, T2DM diagnoses were based on admissions to healthcare facilities in this cohort. As a result, some diagnoses might have been missed, particularly among individuals who do not regularly attend clinics. Second, while the FINDRISC score was originally developed to predict a 10-year risk of diabetes, our study only evaluated a 6-year time frame. This shorter assessment period may have influenced the accuracy of risk predictions. Furthermore, the TurCDRF study did not collect information on second-degree family history, which may have led to the misclassification of individuals with a second-degree family history of diabetes. As a result, we may have underestimated the impact of family history on diabetes risk in the study population. Lastly, the study does not account for genetic predisposition to diabetes. Incorporating genome-wide association studies in future research could provide more personalized and accurate diabetes risk predictions.

## Conclusion

In conclusion, the assessment of the FINDRISC score’s performance in the Turkish population has confirmed its effectiveness in predicting T2DM. Additionally, the country-specific models exhibited discrimination indexes similar to FINDRISC. Reevaluating and tailoring diabetes risk scores for diverse populations have the potential to improve the accuracy of risk assessment. Consequently, both the FINDRISC score and our models serve as suitable tools for identifying T2DM risk in the Turkish population.

## References

[ref1] American Diabetes Association Professional Practice Committee (2021) 2. Classification and diagnosis of diabetes: standards of medical care in diabetes—2022. Diabetes Care 45(Supplement_1), S17–S38. Available at 10.2337/dc22-S002 34964875

[ref2] Fagg J and Valabhji J (2019) How do we identify people at high risk of type 2 diabetes and help prevent the condition from developing? Diabetic Medicine 36, 316–325. Available at 10.1111/dme.13867 30466172 PMC6590463

[ref3] Franciosi M , De Berardis G , Rossi MC , Sacco M , Belfiglio M , Pellegrini F , Tognoni G , Valentini M , Nicolucci A and IGLOO Study Group (2005) Use of the diabetes risk score for opportunistic screening of undiagnosed diabetes and impaired glucose tolerance: the IGLOO (Impaired glucose tolerance and long-term outcomes observational) study. Diabetes Care 28, 1187–1194. Available at 10.2337/diacare.28.5.1187 15855587

[ref4] Gabriel R , Acosta T , Florez K , Anillo L , Navarro E , Boukichou N , Acosta-Reyes J , Barengo NC , Lindström J , Tuomilehto JO and Aschner P (2021) Validation of the Finnish type 2 diabetes risk score (FINDRISC) with the OGTT in health care practices in Europe. Diabetes Research and Clinical Practice 178, 108976. Available at 10.1016/j.diabres.2021.108976 34302911

[ref5] Gao WG , Dong YH , Pang ZC , Nan HR , Wang SJ , Ren J , Zhang L , Tuomilehto J and Qiao Q (2010) A simple Chinese risk score for undiagnosed diabetes. Diabetic Medicine 27, 274–281. Available at 10.1111/j.1464-5491.2010.02943.x 20536489

[ref6] GBD 2021 Diabetes Collaborators (2023) Global, regional, and national burden of diabetes from 1990 to 2021, with projections of prevalence to 2050: a systematic analysis for the global burden of disease study 2021. Lancet (London, England) 402, 203–234. Available at 10.1016/S0140-6736(23)01301-6 37356446 PMC10364581

[ref7] Glümer C , Borch-Johnsen K and Colagiuri S (2005) Can a screening programme for diabetes be applied to another population? Diabetic Medicine: A Journal of the British Diabetic Association 22, 1234–1238. Available at 10.1111/j.1464-5491.2005.01641.x 16108854

[ref8] Hellgren MI , Petzold M , Björkelund C , Wedel H , Jansson PA and Lindblad U (2012) Feasibility of the FINDRISC questionnaire to identify individuals with impaired glucose tolerance in Swedish primary care. A cross-sectional population-based study. Diabetic Medicine 29, 1501–1505. Available at 10.1111/j.1464-5491.2012.03664.x 22443428

[ref9] Jølle A , Midthjell K , Holmen J , Carlsen SM , Tuomilehto J , Bjørngaard JH and Åsvold BO (2019) Validity of the FINDRISC as a prediction tool for diabetes in a contemporary Norwegian population: a 10-year follow-up of the HUNT study. BMJ Open Diabetes Research & Care 7, e000769. Available at 10.1136/bmjdrc-2019-000769 PMC6887494 31803483

[ref10] Kara F , Keskinkilic B , Ekinci B , Altunay Ozkan Z , Sarioglu G , Arikan A and Navruz Kapusuz A (eds) (2021) Noncommunicable diseases and risk factors cohort study in Turkey. T.C. Ministry of Health. Available at https://hsgm.saglik.gov.tr/tr/kronikhastaliklar-haberler/turki-ye-bulasici-olmayan-hastaliklar-ve-ri-sk-faktorleri-kohort-calismasi.html (accessed 18 March 2022).

[ref11] Kposowa AJ , Aly Ezzat D and Breault K (2021) Diabetes mellitus and marital status: evidence from the National Longitudinal Mortality Study on the effect of marital dissolution and the death of a spouse. International Journal of General Medicine 14, 1881–1888. Available at 10.2147/IJGM.S307436 34040420 PMC8139724

[ref12] Lim HM , Chia YC and Koay ZL (2020) Performance of the Finnish diabetes risk score (FINDRISC) and Modified Asian FINDRISC (ModAsian FINDRISC) for screening of undiagnosed type 2 diabetes mellitus and dysglycaemia in primary care. Primary Care Diabetes 14, 494–500. Available at 10.1016/j.pcd.2020.02.008 32156516

[ref13] Lindström J and Tuomilehto J (2003) The diabetes risk score: a practical tool to predict type 2 diabetes risk. Diabetes Care 26, 725–731. Available at 10.2337/diacare.26.3.725 12610029

[ref14] Makrilakis K , Liatis S , Grammatikou S , Perrea D , Stathi C , Tsiligros P and Katsilambros N (2011) Validation of the Finnish diabetes risk score (FINDRISC) questionnaire for screening for undiagnosed type 2 diabetes, dysglycaemia and the metabolic syndrome in Greece. Diabetes & Metabolism 37, 144–151. Available at 10.1016/j.diabet.2010.09.006 21144787

[ref15] Mavrogianni C , Lambrinou CP , Androutsos O , Lindström J , Kivelä J , Cardon G , Huys N , Tsochev K , Iotova V , Chakarova N and Rurik I (2019) Evaluation of the Finnish diabetes risk score as a screening tool for undiagnosed type 2 diabetes and dysglycaemia among early middle-aged adults in a large-scale European cohort. The Feel4Diabetes-study. Diabetes Research and Clinical Practice 150, 99–110. Available at 10.1016/j.diabres.2019.02.017 30796939

[ref16] Omech B , Mwita JC , Tshikuka JG , Tsima B , Nkomazna O and Amone-P’Olak K (2016) Validity of the Finnish diabetes risk score for detecting undiagnosed type 2 diabetes among general medical outpatients in Botswana. Journal of Diabetes Research 2016, 4968350. Available at 10.1155/2016/4968350 27738638 PMC5055990

[ref17] Rintamäki R , Rautio N , Peltonen M , Jokelainen J , Keinänen-Kiukaanniemi S , Oksa H , Saaristo T , Puolijoki H , Saltevo J , Tuomilehto J and Uusitupa M (2021) Long-term outcomes of lifestyle intervention to prevent type 2 diabetes in people at high risk in primary health care. Primary Care Diabetes 15, 444–450. Available at 10.1016/j.pcd.2021.03.002 33771515

[ref18] Saaristo T , Peltonen M , Lindström J , Saarikoski L , Sundvall J , Eriksson JG and Tuomilehto J (2005) Cross-sectional evaluation of the Finnish diabetes risk score: a tool to identify undetected type 2 diabetes, abnormal glucose tolerance and metabolic syndrome. Diabetes and Vascular Disease Research 2, 67–72. Available at 10.3132/DVDR.2005.011 16305061

[ref19] Saaristo T , Moilanen L , Korpi-Hyövälti E , Vanhala M , Saltevo J , Niskanen L , Jokelainen J , Peltonen M , Oksa H , Tuomilehto J and Uusitupa M (2010) Lifestyle intervention for prevention of type 2 diabetes in primary health careOne-year follow-up of the Finnish National Diabetes Prevention Program (FIN-D2D). Diabetes Care 33, 2146–2151. Available at 10.2337/dc10-0410 20664020 PMC2945150

[ref20] Safiri S , Karamzad N , Kaufman JS , Bell AW , Nejadghaderi SA , Sullman MJ , Moradi-Lakeh M , Collins G and Kolahi AA (2022) Prevalence, deaths and disability-adjusted-life-years (DALYs) due to type 2 diabetes and its attributable risk factors in 204 countries and territories, 1990–2019: results from the global burden of disease study 2019. Frontiers in Endocrinology 13, 838027. Available at https://www.frontiersin.org/articles/10.3389/fendo.2022.838027 (accessed 22 August 2023). 35282442 10.3389/fendo.2022.838027 PMC8915203

[ref21] Salinero-Fort MA , Burgos-Lunar C , Lahoz C , Mostaza JM , Abánades-Herranz JC , Laguna-Cuesta F , Estirado-de Cabo E , García-Iglesias F , González-Alegre T , Fernández-Puntero B and Montesano-Sánchez L (2016) Performance of the Finnish diabetes risk score and a simplified Finnish diabetes risk score in a community-based, cross-sectional programme for screening of undiagnosed type 2 diabetes mellitus and Dysglycaemia in Madrid, Spain: the SPREDIA-2 study. Plos One 11, e0158489. Available at 10.1371/journal.pone.0158489 27441722 PMC4956208

[ref22] Sezer Ö , Lafçi NÖ , Korkmaz S and Dağdeviren HN (2021) Prediction of a 10-year risk of type 2 diabetes mellitus in the Turkish population. Medicine 100, e27721. Available at 10.1097/MD.0000000000027721 34871266 PMC8568466

[ref23] Soriguer F , Valdés S , Tapia MJ , Esteva I , Ruiz de Adana MS , Almaraz MC , Morcillo S , Rodríguez F and Rojo-Martinez G (2012) [Validation of the FINDRISC (FINnish Diabetes RIsk SCore) for prediction of the risk of type 2 diabetes in a population of southern Spain. Pizarra Study]. Medicina Clinica 138, 371–376. Available at 10.1016/j.medcli.2011.05.025 21939990

[ref24] Stern MP , Williams K and Haffner SM (2002) Identification of persons at high risk for type 2 diabetes mellitus: do we need the oral glucose tolerance test? Annals of Internal Medicine 136, 575–581. Available at 10.7326/0003-4819-136-8-200204160-00006 11955025

[ref25] Štiglic G , Fijačko N , Stožer A , Sheikh A and Pajnkihar M (2016) Validation of the Finnish diabetes risk score (FINDRISC) questionnaire for undiagnosed type 2 diabetes screening in the Slovenian working population. Diabetes Research and Clinical Practice 120, 194–197. Available at 10.1016/j.diabres.2016.08.010 27592167

[ref26] Tankova T , Chakarova N , Atanassova I and Dakovska L (2011) Evaluation of the Finnish diabetes risk score as a screening tool for impaired fasting glucose, impaired glucose tolerance and undetected diabetes. Diabetes Research and Clinical Practice 92, 46–52. Available at 10.1016/j.diabres.2010.12.020 21242013

[ref27] Traoré S , Paré BC , Dabourou DL , Guira O , Sagna Y , Kamouni JP , Zoungrana L , Bognounou R , Tiéno H and Drabo YJ (2021) Performance of the Finnish diabetes risk score (FINDRISC) in the identification of Dysglycemia in an urban population in Ouagadougou (Burkina Faso). Open Journal of Internal Medicine 11, 39–54. Available at 10.4236/ojim.2021.112003

[ref28] Ünal B , Ergor G , Dinc G , Kalaca S and Sozman K (2013) Chronic diseases and risk factors survey in Turkey. T.C. Ministry of Health. Available at https://ekutuphane.saglik.gov.tr/Yayin/463 (accessed 30 March 2023).

[ref29] Vazquez G , Duval S , Jacobs Jr DR and Silventoinen K (2007) Comparison of body mass index, waist circumference, and waist/hip ratio in predicting incident diabetes: a meta-analysis. Epidemiologic Reviews 29, 115–128. Available at 10.1093/epirev/mxm008 17494056

[ref30] Zhang L , Zhang Z , Zhang Y , Hu G and Chen L (2014) Evaluation of Finnish diabetes risk score in screening undiagnosed diabetes and prediabetes among U.S. adults by gender and race: NHANES 1999–2010. Plos One 9, e97865. Available at 10.1371/journal.pone.0097865 24852786 PMC4031122

